# Cerebrospinal fluid and blood Aβ levels in Down syndrome patients with and without dementia: a meta-analysis study

**DOI:** 10.18632/aging.102560

**Published:** 2019-12-20

**Authors:** Yang Du, Lei Chen, Yuguo Jiao, Yong Cheng

**Affiliations:** 1 Center on Translational Neuroscience, College of Life and Environmental Sciences, Minzu University of China, Beijing 100081, China

**Keywords:** dementia, Down syndrome, amyloid beta, cerebrospinal fluid, blood

## Abstract

Abnormal β-amyloid (Aβ) levels were found in patients with Down syndrome (DS). However, Aβ levels in patients with DS and DS with dementia (DSD) vary considerably across studies. Therefore, we performed a systematic literature review and quantitatively summarized the clinical Aβ data on the cerebrospinal fluid (CSF) and blood of patients with DS and those with DSD using a meta-analytical technique. We performed a systematic search of the PubMed and Web of Science and identified 27 studies for inclusion in the meta-analysis. Random-effects meta-analysis indicated that the levels of blood Aβ_1-40_ and Aβ_1-42_ were significantly elevated in patients with DS compared with those in healthy control (HC) subjects. In contrast, there were no significant differences between patients with DS and those with DSD in the blood Aβ_1-40_ and Aβ_1-42_ levels. The CSF Aβ_1-42_ levels were significantly decreased in patients with DS compared to those in HC subjects. Further, CSF Aβ_1-42_ levels were significantly decreased in patients with DSD compared to those with DS, with a large effect size. Taken together, our results demonstrated that blood Aβ_1-40_ and Aβ_1-42_ levels were significantly increased in patients with DS while CSF Aβ_1-42_, but not Aβ_1-40_ levels were significantly decreased in patients with DS.

## INTRODUCTION

It is well known that patients with Down syndrome (DS), which is a common chromosomal abnormality disease caused by the presence of an extra copy of chromosome 21, have an increased risk of developing early-onset Alzheimer's disease (AD) [1, 2]. The increased risk of AD in patients with DS is thought to be caused by the triplication and overexpression of the gene for the amyloid precursor protein (APP), located on chromosome 21, leading to altered production, aggregation, and deposition of amyloid beta-peptide (Aβ) in the brains of patients with DS [3, 4].

Due to the pathological role of Aβ in the onset and progression of AD, a large number of studies have analyzed blood and cerebrospinal fluid (CSF) Aβ levels in patients with late-onset AD in the general population. Although the results of these studies have been inconsistent, a high-profile systematic review and meta-analysis concluded that patients with AD did not have significantly altered blood Aβ_1-42_ and Aβ_1-40_ levels compared to those in healthy control (HC) subjects. Further, it indicated that the CSF Aβ_1-42_ levels were consistently reduced in the patients with AD patients compared to in HC subjects and suggested that CSF Aβ_1-42_ is a good biomarker for AD diagnosis [5]. Some studies have reported that patients with DS have higher blood Aβ_1-42_ and Aβ_1-40_ levels than those in HC subjects [6–10] while other studies did not find a significant difference in the Aβ_1-42_ levels between patients and HC subjects [11–13]. Blood Aβ_1-42_ and/or Aβ_1-40_ levels seem to alter with age and have been associated with gender in some studies [8, 9, 14] but not in others [15, 16]. Further, there have been inconsistent findings on changes in blood Aβ_1-42_ and Aβ_1-40_ levels after dementia onset [17–19]. Additionally, there have been inconsistent results regarding CSF Aβ_1-42_ and Aβ_1-40_ levels in patients with DS with dementia (DSD) or without dementia [20, 21].

Given the inconsistent findings, there is a need for a meta-analysis of these studies. Therefore, we performed a systematic review and meta-analysis to analyze aberrations in peripheral blood and CSF Aβ_1-42_ and Aβ_1-40_ levels in patients with DS and those with DSD. Further, we evaluated several potential moderators that contribute to the between-study heterogeneity.

## RESULTS

### Blood Aβ_1-42_ and Aβ_1-40_ levels in patients with DS and those with DSD

**Figure 1:**
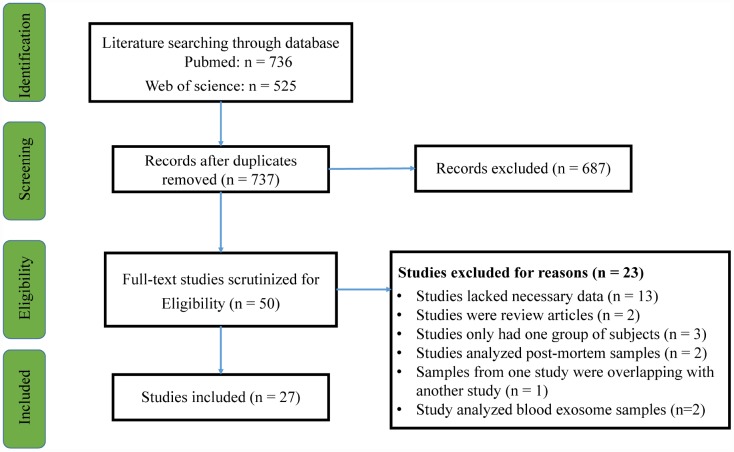
**PRISMA flowchart of the literature search.**

**Figure 2:**
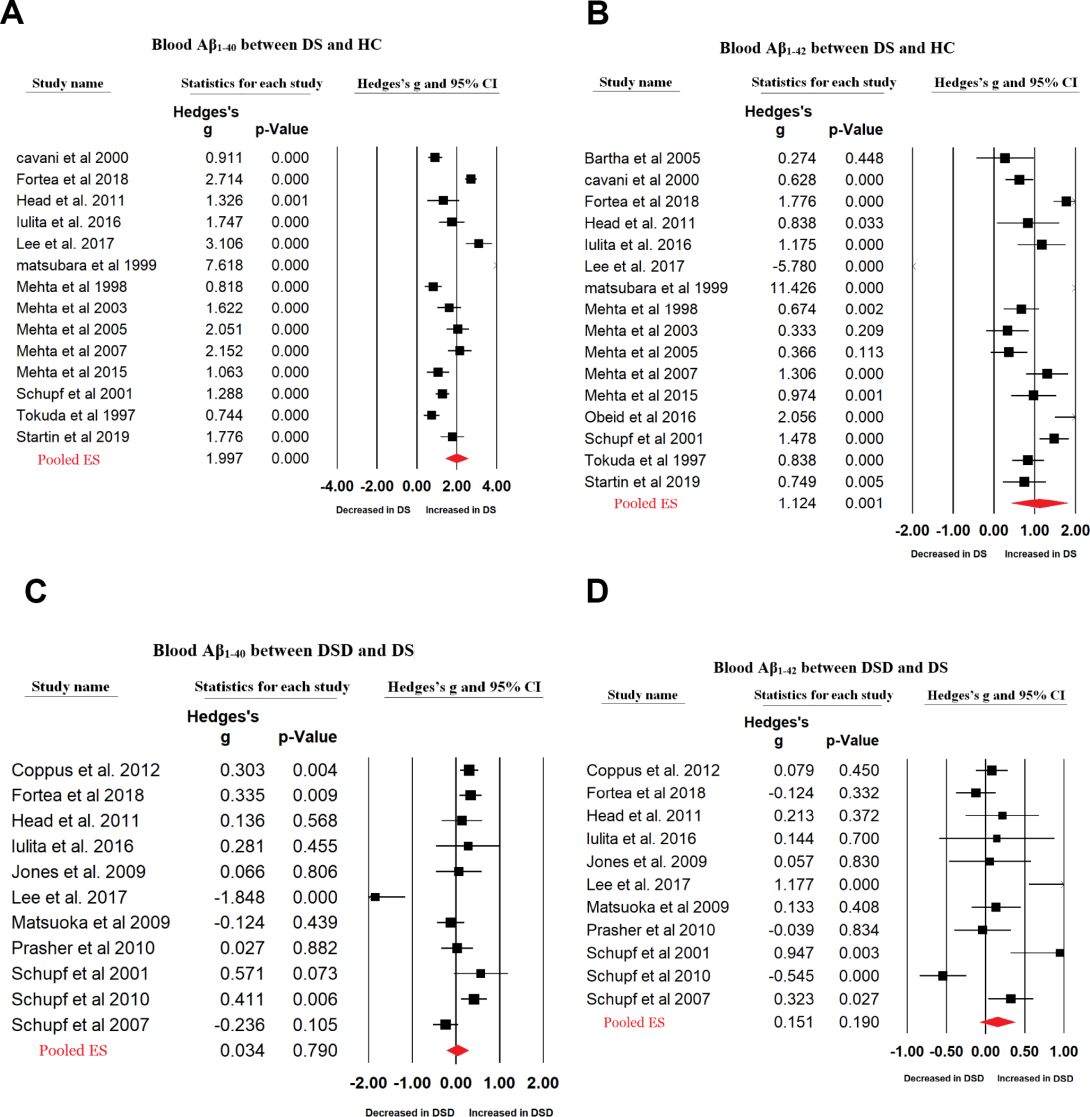
Forest plot for random-effects meta-analysis on difference in blood Aβ_1-40_ (**A**) and Aβ_1-42_ (**B**) concentrations between DS patients and HC subjects; blood Aβ_1-40_ (**C**) and Aβ_1-42_ (**D**) concentrations between DSD and DS patients. DS, Down syndrome. DSD, Down syndrome with dementia. HC, healthy control. CI, confidence interval.

27 articles were included in the current meta-analysis (Figure 1). First, we compared the peripheral blood Aβ_1-42_ and Aβ_1-40_ levels between patients with DS and HC subjects. For Aβ_1-40,_ we used data extracted from 14 studies that included 1440 individuals while for Aβ_1-42_, we used data extracted from 17 studies that included 1587 individuals. Random-effects meta-analysis showed that patients with DS had significantly increased blood Aβ_1-40_ (Hedges’ g = 1.997, 95% CI = 1.422 to 2.571, P < 0.001) and Aβ_1-42_ levels (Hedges’ g = 1.104, 95% CI = 0.445 to 1.763, P = 0.001) compared with HC subjects (Figure 2A, 2B). Sensitivity analysis showed that the significant associations between blood Aβ levels and DS were not affected by one particular study. However, we found significant heterogeneity among studies comparing blood Aβ_1-40_ (Q = 248.253, d.f. = 13, I^2^ = 94.763, P < 0.001) and Aβ_1-42_ levels (Q = 475.084, d.f. = 16, I^2^ = 96.632, P < 0.001) between patients with DS and HC subjects. Further, meta-analysis of the blood Aβ_1-42_/Aβ_1-40_ ration reported in patients with DS by 5 studies encompassing 424 individuals revealed no significant difference between patients with DS and HC subjects (Hedges’ g = -0.830, 95% CI = -1.919 to 0.259, P = 0.135, Supplementary Figure 1A).

Next, we compared the peripheral blood Aβ_1-42_ and Aβ_1-40_ levels between patients with DS and those with DSD using data extracted from 11 studies including 1771 individuals. Random-effects meta-analysis indicated no significant difference in the blood Aβ_1-40_ (Hedges’ g = 0.034, 95% CI = -0.218 to 0.286, P = 0.790) and Aβ_1-42_ levels (Hedges’ g = 0.151, 95% CI = -0.075 to 0.378, P = 0.190) between patients with DS and those with DSD (Figure 2C, 2D). Sensitivity analysis showed that the results for Aβ_1-42_, but not Aβ_1-40_, were influenced by one particular study (Supplementary Figure 2B, 2C). There was significant heterogeneity between studies analyzing blood Aβ_1-40_ (Q = 52.125, d.f. = 10, I^2^ = 80.815, P < 0.001) and Aβ_1-42_ levels (Q = 42.055, d.f. = 10, I^2^ = 76.222, P < 0.001) in patients with DS and those with DSD. Further, analysis of data extracted from 5 studies encompassing 886 individuals indicated no significant difference in the blood Aβ_1-42_/ Aβ_1-40_ ratio between patients with DSD and those with DS (Hedges’ g = 0.029, 95% CI = -0.458 to 0.516, P = 0.907) (Supplementary Figure 1D).

### CSF Aβ_1-42_ and Aβ_1-40_ levels in patients with DS and those with DSD

**Figure 3:**
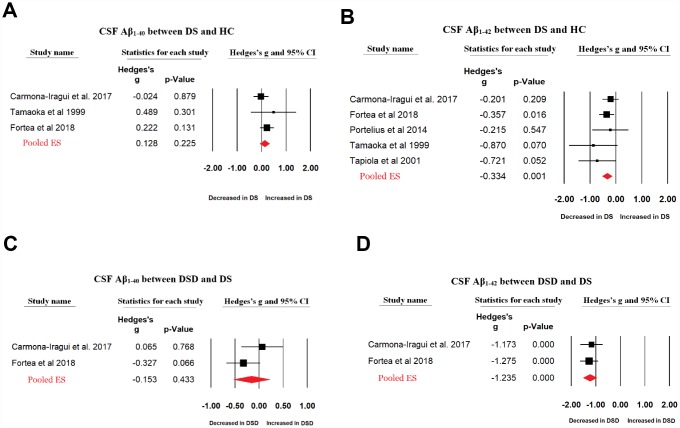
Forest plot for random-effects meta-analysis on difference in CSF Aβ_1-40_ (**A**) and Aβ_1-42_ (**B**) concentrations between DS patients and HC subjects; CSF Aβ_1-40_ (**C**) and Aβ_1-42_ (**D**) concentrations between DSD and DS patients. CSF, Cerebrospinal fluid. DS, Down syndrome. DSD, Down syndrome with dementia. HC, healthy control. CI, confidence interval.

Fewer data were available for CSF Aβ levels in patients with DS and those with DSD. Random-effects meta-analysis did not show a significant difference between patients with DS and HC subjects in the CSF Aβ_1-40_ levels (3 studies, Hedges’ g = 0.128, 95% CI = -0.079 to 0.336, P = 0.226) while CSF Aβ_1-42_ levels were significantly decreased in patients with DS compared with those in HC subjects (5 studies, Hedges’ g = -0.336, 95% CI = -0.530 to -0.143, P = 0.001). In addition, compared with patients with DS, random-effects meta-analysis showed that CSF Aβ_1-42_ levels were significantly decreased in patients with DSD (2 studies, Hedges’ g = -1.235, 95% CI = -1.523 to -0.946, P < 0.001, Figure 3) with a large ES, but not CSF Aβ_1-40_ levels (Hedges’ g = -0.153, 95% CI = -0.535 to 0.229, P = 0.433) (Figure 3). There were no between-study heterogeneities in the studies analyzing CSF Aβ_1-40_ and Aβ_1-42_ levels.

### Investigation of heterogeneity

Next, we investigated the potential sources that influenced the observed heterogeneity and analyzed the studies comparing the blood Aβ_1-40_ and Aβ_1-42_ levels in patients with DS and HC subjects. First, we performed sub-group analysis based on age and the patients were classified into two groups as follows: old (age above 45 years old) and young group (age below 45 years old). Compared with HC subjects, blood Aβ_1-40_ (4 studies, Hedges’ g = 1.331, 95% CI = 1.077 to 1.585, P < 0.001) and Aβ_1-42_ (4 studies, Hedges’ g = 1.065, 95% CI= 0.676 to 1.455, P <0.001) levels were significantly increased in the old group of patient with DS with reduced between-study heterogeneities for both Aβ_1-40_ (Q_3_ = 3.041, I^2^ = 1.338, P = 0.385) and Aβ_1-42_ (Q_3_ = 6.56, I^2^= 54.313, P = 0.087). In contrast, compared with HC subjects, blood Aβ_1-40_ levels were significantly increased in the young group of patients with DS (9 studies, Hedges’ g = 1.750, 95% CI = 1.145 to 2.355, P < 0.001) but not Aβ_1-42_ levels (12 studies, Hedges’ g = 0.443, 95% CI = -0.200 to 1.087, P = 0.177) with between-study heterogeneities remaining high for both Aβ_1-40_ (Q_8_ = 122.948, I^2^ = 93.493, P < 0.001) and Aβ_1-42_ (Q_11_ = 235.814, I^2^ = 95.759, P < 0.001).

Next, we performed meta-regression analyses to assess whether continuous variables, including gender (proportion of males), sample size, and publication year, could explain the between-study heterogeneity. We found that gender, sample size, and publication year did not significantly affect the outcomes of the meta-analysis comparing blood Aβ_1-40_ and Aβ_1-42_ levels between patients with DS and HC subjects (P > 0.05 in all the analyses).

### Publication bias

**Figure 4:**
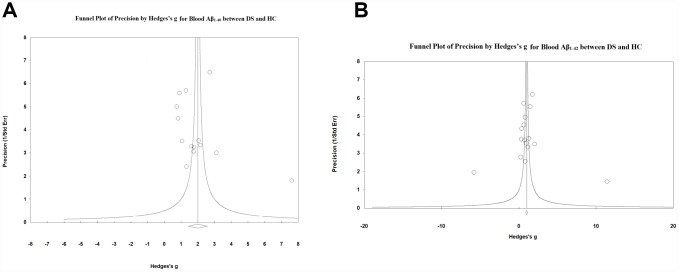
Visual inspection of funnel plots suggested no significant publication bias among studies comparing (**A**) blood Aβ1-40 and (**B**) Aβ1-42 levels between DS patients and HC subjects.

Visual inspection of funnel plots suggested no significant publication bias among studies comparing blood Aβ_1-40_ (Figure 4A) and Aβ_1-42_ (Figure 4B) levels between patients with DS and HC subjects, which was confirmed by Egger’s test (P > 0.05). Further, we used the classic fail-safe N to evaluate potential publication bias and found that 2499 missing studies on blood Aβ_1-40_ and 1027 missing studies on blood Aβ_1-42_ would be required to make p > 0.05, indicating that the significant differences in the blood Aβ levels between patients with DS and HC subjects were unlikely caused by publication bias.

## DISCUSSION

To the best of our knowledge, this is the first systematic review and meta-analysis of clinical studies on Aβ levels in patients with DS and those with DSD. We found significantly increased blood Aβ_1-42_ and Aβ_1-40_ levels in the patients with DS compared with those in HC subjects. However, there were no significant differences in the blood Aβ_1-42_ and Aβ_1-40_ levels between patients with DSD and those with DS. Further, we found that CSF Aβ_1-42_, but not Aβ_1-40_, levels were significantly decreased in patients with DS compared to those in HC subjects while CSF Aβ_1-42_ levels were significantly decreased in patients with DSD compared to those in patients with DS. Taken together, the meta-analysis demonstrated that blood Aβ_1-40_ and Aβ_1-42_ levels were significantly increased in patients with DS while CSF Aβ_1-42,_ but not Aβ_1-40_, levels were significantly decreased in patients with DS. These findings may enhance our knowledge of the molecular mechanism underlying the development and/or progression of dementia in patients with DS.

The baseline elevation of blood Aβ_1-40_ and Aβ_1-42_ levels in patients with DS is a reasonable finding given their overexpression of the APP protein. Increased blood Aβ_1-40_ and Aβ_1-42_ levels in patients with DS was reported in all the included studies except that by Lee et al., which reported decreased blood Aβ_1-42_ levels in patients with DS compared with those in HC subjects [17]. Use of different assay types might explain the inconsistent results regarding blood Aβ_1-42_ levels in patients with DS. This is because Lee et al. used a non-ELISA method to measure Aβ_1-42_ levels while the other studies employed ELISA assay to assess Aβ_1-42_ levels. It remains unclear whether increased blood Aβ_1-42_ and Aβ_1-40_ levels in patients with DS are associated with the development of AD pathology. However, the observed non-significant differences in blood Aβ_1-42_ and Aβ_1-40_ levels between patients with DS and those with DSD suggest that blood Aβ is unlikely to be a key factor in the development of dementia in these patients. This is supported by the findings of a previous systematic review and meta-analysis that reported no significant change in blood Aβ_1-42_ and Aβ_1-40_ levels in the general population of patients with AD [5].

Contrastingly to the observed increased blood Aβ levels in patients with DS, CSF Aβ showed a differential expression profile in the patients. The finding of decreased CSF Aβ_1-42_ levels in patients with DS is consistent with previous reports of Aβ plaque formation in the brains of patients with DS without dementia symptoms [22, 23]. It is unknown why Aβ accumulation and deposition does not lead to dementia before middle age in patients with DS. The small ES of decreased CSF Aβ_1-42_ levels in patients with DS implies that Aβ_1-42_ accumulation in the brains of patients with DS was not detrimental enough to cause global cell death in the central nervous system, and thus lead to dementia onset. This is supported by a previous meta-analysis indicated that CSF Aβ_1-42_ levels were significantly associated with AD in the general population with medium ES [5]. In addition, patients with DSD showed significantly decreased CSF Aβ_1-42_ levels compared with those in patients with DS and with a large ES. This suggests a significant accumulation of Aβ_1-42_ in the brains of patients with DSD, which might explain the early onset of dementia in patients with DS. In contrast to the decreased CSF Aβ_1-42_ levels in patients with DS and those with DSD, there was no significant difference in the CSF Aβ_1-40_ levels among these patients. These results are reasonable since Aβ_1-42_ is the major form of Aβ aggregated and deposited in the brains of patients with DS.

We found significantly increased blood Aβ_1-40_ and Aβ_1-42_ levels in patients with DS and significantly decreased CSF Aβ_1-42_, but not Aβ_1-40_, levels in patients with DS. However, this meta-analysis had several limitations. First, there was a small number of studies analyzing CSF Aβ levels in patients with DS; therefore, future studies are necessary to strengthen our conclusions. Notably, CSF Aβ_1-42_ levels were significant reduced in patients with DSD compared with those in patients with DS and with a large ES, suggesting that CSF Aβ_1-42_ might be a biomarker for the prediction and/or diagnosis of dementia in patients with DS. Future studies are required to validate this hypothesis. Second, it is unclear how age affected the between-study heterogeneity in the meta-analysis of blood Aβ levels. It is possible that the low between-study heterogeneities observed in the old group were due to the small number of studies measuring blood Aβ_1-42_ and Aβ_1-40_ levels in this sub-group. Third, despite exhaustively searching PubMed and Web of Science, we might have missed some eligible studies. However, our analyses suggested that publication bias was unlikely to affect the outcomes of our meta-analysis, indicating the robustness of our findings.

## MATERIALS AND METHODS

We used the guidelines recommended by the Preferred Reporting Items for Systematic reviews and Meta-Analysis (PRISMA) statement to perform this meta-analysis [24].

### Search strategy and study selection

Two investigators independently performed a systematic review of English-language articles from the PubMed and Web of Science databases through April 2019. We searched the databases without year limitation and used the following search terms: (Down syndrome) AND (amyloid-beta OR Abeta OR Aβ). We included studies that compared circulating Aβ_1-42_ and/or Aβ_1-40_ levels between patients with DS and HC subjects or between patients with DS and those with DSD. The original search identified a total of 736 publications from PubMed and 525 publications from Web of Science. After reviewing titles and abstracts, 50 full-text articles were identified and assessed for quantitative analysis eligibility. Among the 50 articles, 23 articles were excluded for the following reasons: 13 lacked the necessary data, 2 were review articles, 3 only had one subject group, 2 analyzed post-mortem samples, 2 analyzed blood exosome samples, and 1 had overlapping samples with another study. Thus, we included a final 27 articles in the current meta-analysis [6–21, 25–35] (Flowchart see Figure 4).

### Data extraction

We extracted data on sample size, mean Aβ_1-42_ and Aβ_1-40_ levels, Aβ_1-42_/Aβ_1-40_ ratio, standard deviation (s.d), and p-values as primary outcomes. We also extracted data on the average ages, gender distribution, sample sources (blood or cerebrospinal fluid), assay type, region of studies, and publication year. Two investigators independently extracted the data and any inconsistencies in the extracted data were settled by discussion. Supplementary Table 1 summarizes the demographic and clinical characteristics in the included studies.

### Statistical analysis

The Comprehensive Meta-Analysis Version 2 software (Biostat, Englewood, NJ, USA) was used for the meta-analysis. We primarily used the sample sizes, mean Aβ levels, and s.d. to generate effective sizes (ESs). In some studies, sample sizes and P values were used to generate ESs as mean Aβ levels and the s.d. were not available. We calculated the ESs as the standardized between-group mean difference in the Aβ_1-42_ and Aβ_1-40_ levels and converted to Hedge's g, which provides an unbiased ES adjusted for sample size. The 95% confidence interval (CI) was used to assess statistical differences in the pooled ES. We chose random-effects models for the meta-analysis since we hypothesized that within-study and between-study moderators would result in differences in the true ES [24]. We performed sensitivity analysis by removing one study at a time to test whether a particular study significantly affected the outcomes of the meta-analysis.

Statistical differences in the across-study heterogeneity were assessed using Cochran Q test [36] with the statistical significance set at P value < 0.1. The across-study inconsistency was determined by the I^2^ index to evaluate the impact of heterogeneity. An I^2^ of 0.25, 0.50, and 0.75 suggested small, moderate, and high levels of heterogeneity, respectively [37]. Next, we used unrestricted maximum-likelihood random-effects meta-regression of the ES [38] to assess whether potential moderators including mean age, gender distribution (male proportion), and publication year affected the ES. Funnel plots generated by plotting the ES against the study precision (inverse of standard error) were used for visual inspection of publication bias. A statistical test for significance of publication bias was determined by Egger’s test [39], which assesses the degree of funnel plot asymmetry. Classic fail-safe N, which is an analysis of the number of missing (unpublished) studies that allow the observed P value to reach > 0.05, was also used to investigate publication bias.

All statistical significances were set at P value > 0.05 except where otherwise noted.

## Supplementary Material

Supplementary Figure 1

Supplementary Table 1
