# Supplementary material for: Cerebrospinal fluid and blood Aβ levels in Down syndrome patients with and without dementia: a meta-analysis study
Source: Aging (Albany NY). 2019 Dec 20;11(24):12202–12. doi: 10.18632/aging.102560 (PMC6949072; doi:10.18632/aging.102560)
Supplement: Supplementary Figure 1 [file aging-11-102560-s002..pdf]

SUPPLEMENTARY FIGURE

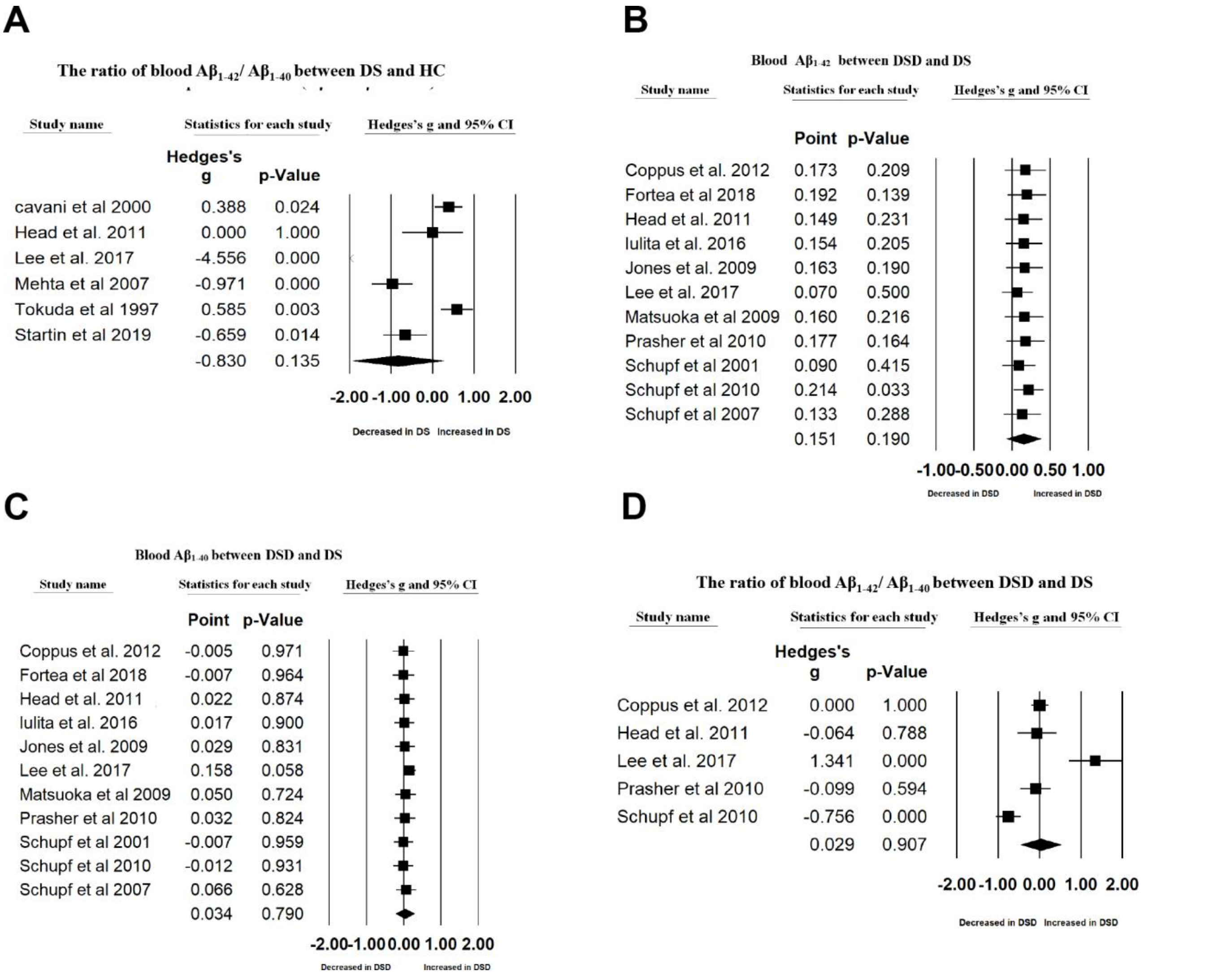

Supplementary Figure 1. The ratio of blood Ap1-42/ Ap1-40 between (**A**) DS patients and HC subjects and (**D**) DSD and DS patients. Sensitivity analysis for (**B**) blood Ap$_{1-42}$ levels and (**C**) blood Ap$_{1-42}$ levels between DSD and DS patients. DS, Down syndrome. DSD, Down syndrome with dementia. HC, healthy control. CI, confidence interval.
