# Supplementary material for: Cerebrospinal fluid and blood Aβ levels in Down syndrome patients with and without dementia: a meta-analysis study
Source: Aging (Albany NY). 2019 Dec 20;11(24):12202–12. doi: 10.18632/aging.102560 (PMC6949072; doi:10.18632/aging.102560)
Supplement: Supplementary Table 1 [file aging-11-102560-s001..pdf]

SUPPLEMENTARY TABLE

Supplementary Table 1. Papers included in the meta-analysis on CSF and blood Ap levels in Down syndrome.

Study/year	Ap measured	Country	Samples DS/DSD/Control	Gender (% Male) DS/DSD/Control	Mean age DS/DSD/Control	Sample source	Assay type
Bartha et al 2005	AP$_{1-42}$	Spain	13/NA/17	NA/NA/NA	0/NA/0	plasma	immunoassay
Carmona-Iragui et al. 2017	AP$_{1-42}$, AP$_{1-40}$	Spain	91/26/68	56/54/35.3	39.6/53.8/54.3	CSF	ELISA
Cavani et al 2000	AP$_{1-42}$, AP$_{1-40}$	Italy	61/NA/77	NA/NA/NA	25.9/NA/32	plasma	ELISA
Conti et al. 2010	AP$_{1-42}$	Italy	24/NA/18	79/NA/61	28.23/NA/28.2	plasma	ELISA
Coppus et al. 2012	AP$_{1-42}$, AP$_{1-40}$	Netherlands	264/141/NA	64/58/NA	50.6/53.6/NA	Plasma	Luminex2000
Fortea et al 2018	AP$_{1-42}$, AP$_{1-40}$	Spain	194/88/67	54/56/30	37/53/52	Plasma	ELISA
			54/40/67	56/60/30	37/53/52	CSF	ELISA
Head et al. 2011	AP$_{1-42}$, AP$_{1-40}$	USA	17/NA/11	52.9/NA/45.5	44.1/NA/46.5	Plasma	ELISA
			26/52/NA	65/50/NA	45.1/50.3/NA	Plasma	ELISA
Iulita et al. 2016	AP$_{1-42}$, AP$_{1-40}$	Italy	21/10/31	48/60/52	34/52/38	Plasma	Multi-Spot V-PLEX Ab Peptide Panel
Jones et al. 2009	AP$_{1-42}$, AP$_{1-40}$	UK	39/21/NA	NA/NA/NA	48.8/54/NA	Plasma	ELISA
Lee et al. 2017	AP$_{1-42}$, AP$_{1-40}$	China	35/16/44	54.3/62.5/68.2	25.6/35.4/38.4	Blood (Serum)	Immunomagnetic ReductionAssay
Bourque et al 1999	AP$_{1-42}$, AP$_{1-40}$	Canada	6/NA/206	NA/NA/NA	16.8/NA/NA	Whole Plasma	ELISA
Matsuoka et al 2009	AP$_{1-42}$, AP$_{1-40}$	USA	148/52/NA	60/64/NA	54.2/56/NA	plasma	ELISA
Mehta et al 1998	AP$_{1-42}$, AP$_{1-40}$	USA	43/NA/43	53/NA/49	44/NA/43	Blood	ELISA
Mehta et al 2003	AP$_{1-42}$, AP$_{1-40}$	USA	28/NA/28	53/NA/53	30/NA/30	Blood	ELISA
Mehta et al 2005	AP$_{1-42}$, AP$_{1-40}$	USA	38/NA/37	50/NA/51	45/NA/44	Blood	ELISA
Mehta et al 2007	AP$_{1-42}$, AP$_{1-40}$	USA	35/NA/34	80/NA/59	7.17/NA/10.71	Blood	ELISA
Mehta et al 2015	AP$_{1-42}$, AP$_{1-40}$	USA	29/NA/26	24/NA/35	48/NA/47	plasma	ELISA
Obeid et al 2016	AP$_{1-42}$	Germany	31/NA/44	54/NA/48	12.1/NA/12.8	Blood	ELISA
Portelius et al 2014	AP$_{1-42}$	Sweden	12/NA/20	50/NA/67	41/NA/40	CSF	ELISA
Prasher et al 2010	AP$_{1-42}$, AP$_{1-40}$	UK	83/44/NA	62.7/68.3/NA	49/56.8/NA	Blood	ELISA
Schupf et al 2001	AP$_{1-42}$, AP$_{1-40}$	USA	97/11/64	NA/NA/NA	51.9/56/51.5	Blood	ELISA
Schupf et al 2010	AP$_{1-42}$, AP$_{1-40}$	USA	164/61/NA	33.5/29.5/NA	50.3/53.7/NA	plasma	ELISA
Tamaoka et al 1999	AP$_{1-42}$/AP$_{1-40}$	Japan	5/NA/34	NA/NA/NA	55.3/NA/67.9	CSF	ELISA
Tapiola et al 2001	AP$_{1-42}$	Finland	12/NA/19	50/NA/58	41/NA/53	CSF	ELISA
Tokuda et al 1997	AP$_{1-42}$, AP$_{1-40}$	Japan	44/NA/66	NA/NA/NA	44.1/NA/44.1	plasma	ELISA
Schupf et al 2007	AP$_{1-42}$, AP$_{1-40}$	USA	130/74/NA	NA/NA/NA	NA/NA/NA	Blood	ELISA
Startin et al 2019	AP$_{1-42}$, AP$_{1-40}$	UK	31/NA/27	71/NA/59.3	46.77/NA/49.26	Blood	ELISA

CSF, Cerebrospinal fluid. DS, Down syndrome. DSD, Down syndrome with dementia. ELISA, enzyme-linked immunosorbent assay.
